# 

**Published:** 1903-04

**Authors:** 


					﻿Vol. XVIII.
APRIL, 1903.
No. 10.
TEXAS
Medical Journal.
IQ
W-
Jo
>4 •
*83
* Im
fe’
B
to
A
Is
I fl
rS •
k‘
I fl
ro
M-*
I 83
1*^
I'O
i «
!<■
if-H
183
£□
r*1M
hO
F'
te
[ 83
>
m-
SUBSCRIPT’dN, $T .OO A YEAR; TN At^A>rCE.1
Single Copies, io Cents;.‘	’
PUBLISHED AT AU8TIN, TEXAS, BY
. F. E. DANIEL, M. D;, ‘
Proprietor.
L ist?rr J<eDic.S6n?u.tlvi: io tn C i / 3lt>!iib i:e; St. Paul Building, 2y) Broadway. New
York City. •	.
Entered at t be Fob toffice at- Austin, Texas, as Second-olaaa Mall Matter.
				

